# Treatment of Middle Third Humeral Shaft Fractures with Anteromedial Plate Osteosynthesis through an Anterolateral Approach

**DOI:** 10.5704/MOJ.1603.007

**Published:** 2016-03

**Authors:** BS Kumar, P Soraganvi, D Satyarup

**Affiliations:** Department of Orthopaedics, PES Institute of Medical Sciences and Research, Kuppam, India

**Keywords:** Humeral shaft fractures, Plate osteosynthesis, Antero-lateral approach

## Abstract

**Background:** Treatment of humeral shaft fractures has been a subject of debate for many decades. Even though a large majority of humeral shaft fractures can be treated by non operative methods, few conditions like open fractures, polytrauma, ipsilateral humeral shaft and forearm fractures require surgical intervention. The goal of treatment of humeral shaft fractures is to establish union with an acceptable humeral alignment and to restore the patient to pre-injury level of function. The objective was to assess the incidence of radial nerve palsy, non-union and mean time required for in anteromedial plate osteosynthesis with anterolateral approach and also to measure the functional outcome of this procedure.

**Method:** A prospective study was conducted in the Department of Orthopaedics, PESIMSR, Kuppam, Andhra Pradesh, from August 2012 to August 2015 with a total of 54 patients who were operated with anteromedial plate osteosynthesis were included in the study. Rodriguez- Merchan criteria was used to grade the functional outcome.

**Results:** Of the 54 patients, 28 (58.85%) were in the age group of 30-40 years. The most common fracture pattern identified was A3 type (48.14%).The mean (± SD) duration of surgery for anteromedial humeral plating was 53 ± 5.00 minutes. The time taken for the fracture to unite was less than 16 weeks in the majority or 50 patients (92.59%). Four (7.40%) patients had delayed union. There was no incidence of iatrogenic radial nerve palsy. Rodriguez – Merchan criteria showed that 37(68.51%) of the patients had good and 12 (22.22%) had excellent functional outcome.

## Introduction

Fractures of humeral shaft are commonly encountered by orthopaedic surgeons, accounting for approximately 3% of all fractures, which result in significant burden to society from lost productivity and wages^1^. The goal of treatment of the humeral shaft fractures is to establish union with an acceptable humeral alignment and to restore the patient to pre-injury level of function^2^. Even though the majority of humeral shaft fractures can be treated by non-operative methods, the advantages of non-surgical approach are that non-union, wound infection and iatrogenic radial nerve palsy are low^3^. However, operative treatment are required in a few conditions like open fractures, poly-trauma, ipsilateral humeral shaft and forearm fractures and in cases in which there is failure to tolerate or maintain alignment of functional brace^4^. Surgical management includes plate osteosynthesis, intra-medullary nailing or external fixation. Plate osteosynthesis remains the gold standard for the operative fixation of humeral shaft fractures despite advances in implant technology^5^. The incidence of postsurgical radial nerve palsy ranges from 0% to 5.1%^6,7^. The causes of radial nerve palsy include manipulation of the nerve during surgery, impingement between fracture fragments, entrapment by fracture callus and tissue scar formation. In order to seek a better surgical approach with minimal complications this study was undertaken in our hospital with the following additional objectives:

1) the incidence of radial nerve palsy and non-union in anteromedial plate osteosynthesis with anterolateral approach;
2) the mean time required for anteromedial plate osteosynthesis
3) the functional outcome of this procedure.

## Materials and Methods

This prospective study was conducted at the Department of Orthopaedics in PES Institute of Medical Science and Research (PESIMSR) Kuppam, Andhra Pradesh, from August 2012 to August 2015. Data was collected from patients through predesigned questionnaire. The data sheet contained information on demographics, side of injury, fracture type, mechanism of injury, type of treatment, duration of surgery, time taken to unite and complications. Written informed consent was obtained from every patient. Data was collected after surgical treatment and on follow up in all patients for complications. A total of 54 patients who were treated with anteromedial plate osteosynthesis through an anterolateral approach were included in the study. Ethical clearance was obtained from the institutional ethics committee. Statistical analysis was done using SPSS 17.0 trial version.

**Fig. 1:**
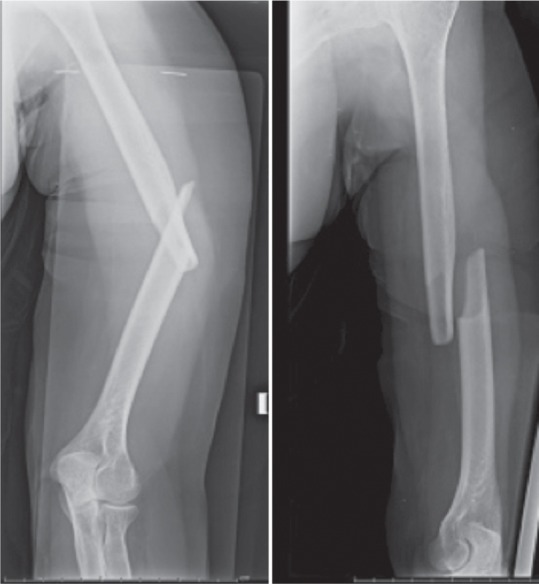
Anteroposterior and lateral radiographs showing fracture middle third shaft of humerus.

Inclusion criteria: Patients with acute middle third humeral fractures treated with anteromedial plate osteosynthesis through an anterolateral approach were included in the study. (Figures: 1a and 1b)

Exclusion criteria: Patients less than 18 years of age, preoperative radial nerve palsy, pathological fracture, associated injuries in the same limb and those lost to follow up were excluded from the study.

## Operative Technique

**Fig. 2:**
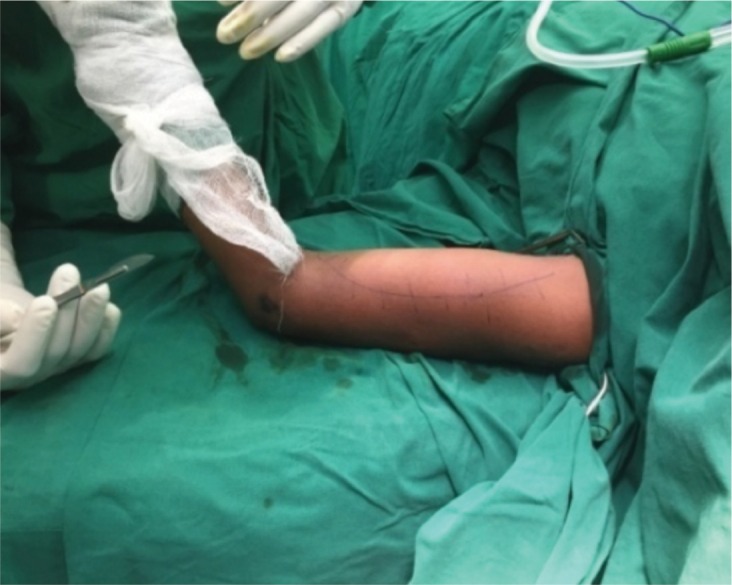
Position of skin incision along the lateral border of biceps.

**Fig. 3a & 3b:**
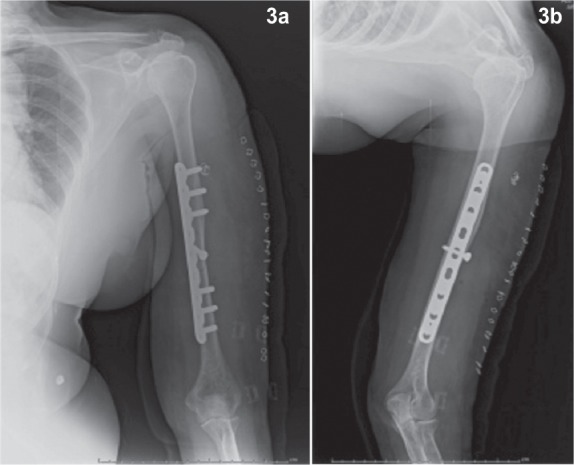
Immediate post operative anteroposterior and lateral radiographs showing Plate position on anteromedial surface of humerus.

The patients were placed on supine position on an operating table with the arm in abduction on arm board. The entire limb was prepared exposing both shoulder and elbow. The humerus middle third was approached by the standard Henry’s approach. The incision was made along the lateral border of biceps (Figure 2) with sufficient length to allow insertion of 8 to 10 hole LCP (Locking Compression Plate) or LCDCP (Limited Contact Dynamic Compression Plate). The space between biceps and brachialis was identified and the musculocutaneous nerve visualized and protected. The biceps was retracted medially and the brachialis muscle was split longitudinally to expose the humerus. The arm was externally rotated to facilitate the visualization of the anteromedial surface of the humerus. Fractures were reduced and fixed with 4.5 mm LCP or LCDCP by the AO principle. Immediate post operative radiograph was taken. (Figures 3a and 3b)

**Fig. 4a & 4b:**
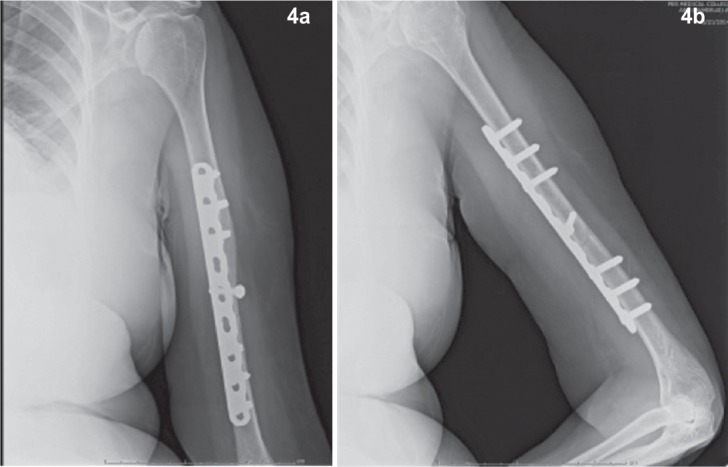
Anteroposterior and lateral radiographs at the end of 12 weeks of follow up:

Active shoulder and elbow range of motion exercises were started on the second post-operative day and sutures were removed on the 10th post-operative day and the patient discharged. Patients were advised to continue movements with arm pouch support and were followed up every month for nine months. During each follow up visit, each case was examined for pain, functional recovery and the progress of fracture healing was radiologically documented. (Figures 4a and 4b) Rodriguez-Merchan criteria was used to grade the functional outcome of the anteromedial plating^8^ [Table:I]. The overall outcome rating of excellent, good, fair and poor was based on scores of shoulder and elbow movements, pain and disability after the surgical procedure.

**Table I:** Criteria for evaluating functional results

Rating	Elbow range of movement	Shoulder range of movement	P value	Pain Disability
Excellent	Extension 5°	Full range of movement	None	None
	Flexion 130°			
Good	Extension 15°	<10% loss of total range of movement	Occasional	Minimum
	Flexion 120°			
Fair	Extension 30°	10–30% loss of total range of movement	With activity	Moderate
	Flexion 110°			
Poor	Extension 40°	>30% loss of total range of movement	Variable	Severe
	Flexion 90°			

## Results

A total of 54 patients were included in the study, the majority of whom were males 41 (75.92%). The age group of the patients ranged from 21 to 65 years, the majority (28 of them or 58.85%) being in the 30 - 40 year age group. (Table II) The majority of the patients (39 or 72% ) were victims of road traffic accidents followed by ten patients (19%) with history of falls from height and five (9%) with history of assault. (Graph I)

**Table II:** Age wise distribution of patients:

Age group of patients (in years)	Number of patients	Percentage
21-30	6	11.11%
31-40	28	51.85%
41-50	13	24.07%
51-60	7	12.96%

**Graph I:**
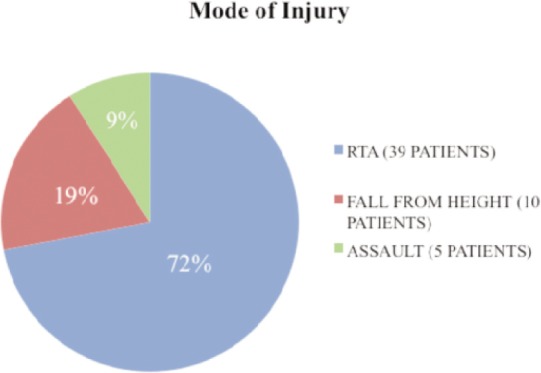
Mode of injury.

The most common fracture pattern identified in this study was A3 type 26 which accounted for 48.14% followed by A2 type 13 (24.07%), B2 type 9 (16.66%) and A1 type which was least , 6 (11.11%). (Graph II) The mean (+ SD) duration of surgery for anteromedial humeral plating was 53 ± 5.00 minutes. The duration for fracture union was less than 16 weeks in the majority in 50 (92.59%) of the patients. (Table III)

**Graph II:**
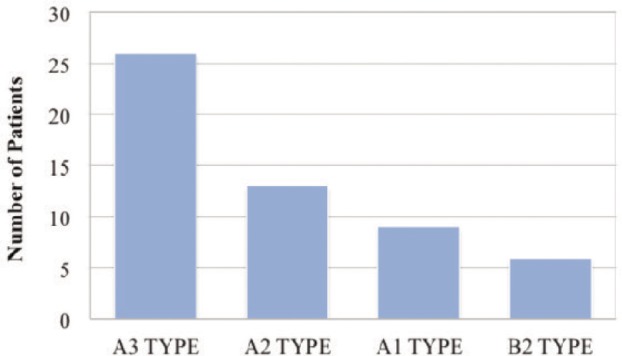
Distribution of Fracture type.

**Table III:** Time taken for union

Time taken for union	No. Of patients	Percentage
<16weeks	50	92.59%
>16 weeks	4	

Of the 54 patients only 4 (7.40%) patients had delayed union. There was no evidence of iatrogenic radial nerve injury. (Table IV) Functional assessment was done at the final follow using the Rodriguez Merchan criteria which showed that 37(68.51%) of the patients had good functional outcome and 12(22.22%) of the patients had excellent outcome. Only 4 (7.40%) of the patients had poor outcome.

**Table IV:** Incidence rate of complications in Anteromedial plating of humerus

Incidence rate in anteromedial humeral plating	Number of patients	Number of patients
Normal	50	92.59%
Delayed union	4	7.40%
Iatrogenic radial nerve palsy	0	0%

**Table V:** Criteria for evaluating functional results

Result	Number of patients	Percentage
Excellent	12	22.2%
Good	37	68.5%
Fair	1	1.8%
Poor	4	7.4%

## Discussion

A few studies have shown that humeral shaft fractures have bimodal distribution. In the elderly, the predominant causes include simple falls or rotational injuries, whereas high energy mechanisms are usually involved in younger patients, as in motor vehicle accidents, assaults, falls from height and throwing injuries^5,9-12^. However, in our study we did not find bimodal distribution and the majority (51.85%) of the patients were in the age group of 31-40 years and mainly of males following road traffic accidents and this was similar to a study from Taiwan^11-13^. This was again contrary to a report from Scotland, which indicated the highest incidence in patients aged over 50 years^19^. Analysis of humeral fractures in general in this study in relation to gender showed that males had a higher incidence than females, as had also been shown from an American study^20^. This might be due to the fact that men are more involved in physically demanding jobs than women. A previous study also showed that men are more involved in road traffic accidents than women^21^.

The most common fracture type seen in our study was A3 type (48.14%) which was similar to other studies from various other countries^10,11,13^. In the present study with anteromedial humeral plating the union rates were high and in majority of the patients time taken for fracture to unite was less than 16 weeks which was similar to a study done in India^1^.

Ivan Kirin *et al* (2011) did both anteromedial and anterolateral plate osteosynthesis by anterolateral approach and the mean operation time for anterolateral plating was 74.61 + 10.74 min. and for anteromedial humeral surface plating 55.45 + 10.56 min^2^.

Mean (+ SD) duration of surgery in our study was 53+5 min which was less then for the anterolateral group and comparable to similar study reported by Ivan *et al* (2011) which signifies that anteromedial humeral plating is less time consuming compared to other surgical approaches.

In our study we did not come across any iatrogenic radial nerve injuries. The literature reported incidence of iatrogenic radial nerve palsy when the plate was placed on antero lateral and posterior surface of humerus was up to 12% (5) and 11.46% (2). One of the main advantage with anteromedial plate osteosynthesis was that there was less iatrogenic radial nerve palsy.

There were four cases of delayed union, but we did not find any obvious cause for the delayed union. Among these fracture three united in three spontaneously and one patient required bone grafting. There was no instances of osteomyelitis or wound infection.

As the anteromedial surface has relatively less muscle attachments, anteromedial humeral approach does not require unnecessary soft tissue dissection or stripping compared to anterolateral humeral plating. A few authors have observed that the main concern regarding plate placement on the anteromedial surface of humerus was damage to the nutrient artery of the humerus^14,15^. As described by Laing,^19^ the displaced fractures at the junction of middle and lower third humerus shaft would probably destroy the main nutrient artery and open reduction of middle third humerus fractures and the damage to the nutrient artery was greatest. The blood supply to the upper end of the distal fragment depends on periosteal vessels and ascending branches from the epicondyles. The proximal fragment is supplied by accessory nutrient arteries and the periosteum. This suggests that anteromedial humeral plating is associated with less surgical complications.

Based on the Rodriguez Merchan criteria, the higher rate of excellent and good results with anteromedial humeral plating seen in our study was also supported by a few other reports^17,18^.

## Conclusion

Most humeral shaft fractures can be treated non-operatively but, when operative indication is indicated we would suggest that the better option would be anteromedial humeral plating with anterolateral approach, as it is less time consuming, has less complications and produces excellent functional outcome. The main concern of iatrogenic injury to radial nerve was not encountered in this procedure in the present study. We finally conclude that anteromedial humeral plating with the anterolateral approach has both better surgical and functional outcome especially in the middle third fracture of the shaft of humerus.

## Recommendations

Further randomised trials should be done to bring out further strong evidence that anteromedial humeral plating with anterolateral approach has both better surgical and functional outcome in - middle third - fracture of the humeral shaft.

**Author’s disclosure statement:** The authors report no actual or potential conflict of interest in relation to this study.
